# Reinforcement of rectal anastomoses with a collagen-based haemostatic patch: a case series report

**DOI:** 10.1093/jscr/rjaa228

**Published:** 2021-04-13

**Authors:** Dan Kornfeld

**Affiliations:** Capio Sankt Görans Hospital, Stockholm, Sweden

## Abstract

In this case series report of 10 colorectal cancer patients, a polyethylene glycol-coated collagen-based haemostatic patch was applied after rectal resection to reinforce rectal anastomoses and reduce anastomotic leakage. Patients underwent rectal resection and anastomoses were stapled in place. The patch—Hemopatch®—was applied to 75% of the anastomotic circumference. The surgeon judged the simplicity of application using a reinforcement of rectal anastomosis score. Mean age of patients was 68.1 (range 50–94) years. The patch was successfully applied in eight patients; in seven patients, patch application was straightforward or only slightly complex, according to the reinforcement of rectal anastomosis score. Seven of eight patients experienced no leakage or signs of stricture 6 weeks post-surgery. All patients underwent radical resection. It is possible to apply Hemopatch® during colorectal surgery. However, the patch application procedure needs to be standardized and efficacy needs to be evaluated by conducting larger clinical studies.

## INTRODUCTION

Rectal surgery has become the predominant technique to prevent the spread of rectal tumours [1]. After excision, the colon is stapled to the anal tissue, forming a rectal anastomosis. However, anastomotic leakage is a major concern, with risk of leakage as high as 24% and increased morbidity between 6% and 22% [1]. Risk factors for leakage include the height of the anastomosis (>6 cm), age and preoperative neoadjuvant therapy (chemo- or radiotherapy) [2], but predicting individual patient risk has proven difficult as few identified risk factors are conditionally independent [3].

A polyethylene glycol (PEG)-coated, collagen-based, haemostatic patch (Hemopatch®, Baxter AG, Vienna, Austria), designed to stop bleeding during surgical procedures and to induce hemostasis [4] could potentially increase mechanical strength around the anastomosis and could offer an alternative treatment strategy. This case series—pilot study aimed to investigate the practicability of applying the haemostatic patch (Hemopatch®), on rectal anastomoses in colorectal cancer patients and to monitor the success of the patch in preventing anastomotic leakage.

The study was approved by the Ethics Committee at the Karolinska Institute, Sweden, according to principles enshrined in International Council for Harmonization Good Clinical Practice and the Declaration of Helsinki. All subjects were informed of the purpose of the study and signed voluntary consent forms before enrolment.

**Table 1:** Cancer characteristics of patients

Sex (male/female)	Cancer type (rectal/sigmoid colon)	Distance from anal verge (cm)	Tumour/node/metastasis score
Female	Rectal	8	T2 N0 M0
Male	Rectal	8	T2 N0 M0
Male	Rectal	12	T3 N0 M0
Male	Sigmoid colon	18	T3 N0 M0
Male	Rectal	11	T3 N0 M0
Male	Rectal	15	T3 N0 M0
Female	Rectal	7	T2 N0 M0
Female	Rectal	12	T3 N0 M1
Male	Rectal	9	T3 N2 M0
Male	Rectal	13	T2 N0 M0

**Table 2:** Frequency of patient outcomes in each RORA score category

RORA score	RORA freq n	Age (years) mean (range)	Neoadjuvant therapy n (%)	Protective ileostomy *n* (%)	Anastomotic leakage n (%)	Radical resection *n* (%)	Stricture *n* (%)
1	2	64 (62–66)	0 (0)	2 (100)	1 (50)	2 (100)	0 (0)
2	5	64.8 (50–78)	1 (20)	3 (60)	0 (0)	5 (100)	0 (0)
3	1	94	0 (0)	1 (100)	0 (0)	1 (100)	0 (0)
X	2	67.5 (55–80)	1 (50)	1 (50)	1^*^(50)	2 (100)	0 (0)

RORA = reinforcement of rectal anastomosis; freq = number of patients in each RORA score category.

RORA scale: 1 = haemostatic patch was easily applied with full visibility; 2 = partial difficulty in extending and inspecting patch placement over the entire anastomosis; 3 = patch applied, inspected and extended with difficulty; X = the patch could not be applied.

^*^This was not a leak, but an abscess.

Eligible for this study eligible were colorectal cancer patients with an estimated height from the anal verge to the anastomosis of 10 cm or less were. Ten patients were thought to be a sufficient number of patients to achieve the study aim. Outcomes measured in this study were the success of reinforcement of the rectal anastomosis (RORA) with the haemostatic patch, anastomotic leakage, radical resection and signs of stricture 6 weeks after patch application.

Patients were enrolled over 13 months, from 18 May 2016 to 13 June 2017. Open surgery was performed in all cases, as no appropriate tool was available to apply the patch in a laparoscopic colorectal setting. During surgery, anastomoses were stapled end to side using the circular end-to-end anastomosis device (Autosuture®; Covidien Sverige AB, Stockholm, Sweden) and a ‘leak test’ was performed by submerging the anastomosis in saline solution while filling the rectum with air. The patch was wrapped around ~75% of the anastomosis using the closed stapler as an anvil. In all cases the patch covered the posterior part of the anastomosis, leaving 25% of the anterior exposed. This approach was taken because most leaks occur in the posterior part of the anastomosis, and leaving 25% exposed reduced the risk of stricture [5]. The surgeon judged how easy it was to expand and attach the patch using an arbitrary scale (RORA score): (i) the patch was easily applied with full visibility; (ii) partial difficulty in extending and inspecting patch placement over the entire anastomosis; (iii) patch applied, inspected and extended with difficulty; (iv) the patch could not be applied.

All patients entered the hospital’s early recovery after surgery program [6]. Postoperative outcome was monitored every 4 h for the first 3 days after surgery using the modified early warning score. Laboratory analyses (C-reactive protein, white blood cell count, haemoglobin, creatinine, electrolytes) were performed on all postoperative days. In addition, nutritional status, urine output, physical examination, temperature, heart rate, respiratory rate, systolic blood pressure and central nervous system status were recorded and scored. Patients were discharged 3–10 days after surgery. All patients were followed up for signs of stricture 6 weeks after surgery.

## CASE REPORT

A total of 17 colorectal cancer patients were screened. Seven patients were excluded as five patients had anastomoses over 10 cm above the anal verge, one patient underwent whole rectum excision and one patient was assigned to robotic rectal surgery at a later date. Ten patients—seven men and three women—met the criteria for inclusion (Table 1). Mean age was 68.1 years (range 50–94 years). Mean body mass index was 25.32 kg/m^2^ (range 17.7–31.1 kg/m^2^). A protective loop ileostomy was performed on 7 of the 10 patients.

The haemostatic patch was applied to the rectal anastomosis in eight patients (Table 2). The patch was applied easily in two patients (RORA score 1), extended and inspected with partial difficulty in five patients (RORA score 2), and applied with great difficulty in one patient (RORA score 3). Two patients did not have a haemostatic patch applied during surgery following a clinical decision by the performing surgeon in the theatre. Only one of the eight patients that had the patch experienced anastomotic leakage. All patients underwent a radical resection, and no patient showed signs of stricture at the 6-week follow-up.

## DISCUSSION

This study has shown that it is possible to apply a haemostatic patch in a colorectal open intraoperative setting. Seven of eight patients experienced no anastomotic leakage or stricture after 6 weeks. However, applying the patch was complex in some cases, suggesting it would be preferable to standardize the procedure even more. This small scale pilot study presents no firm conclusions that can be drawn regarding the ability of the patch to prevent leakage. However, the result is promising and indicates that the use of haemostatic patches in colorectal surgery should be explored further.

The current method used to mitigate anastomotic leakage is a stoma diversion [1]. However, stoma diversions are often permanent, impractical, expensive, and can cause complications [2, 7, 8]. Using a haemostatic patch circumvents these drawbacks. The flexibility and rapid adhesion of Hemopatch® make it potentially suitable for use in colorectal surgery. However interpretation of the practicability of haemostatic patch use has so far been impeded by the nature of studies that have been conducted, which were small in scale and based on *in vitro* and *in vivo* animal studies [9].

This study yielded the need to standardize haemostatic patch application in a reproducible and effective way. The next step would be to conduct a large randomized study specifically designed to evaluate both patient and procedure-related factors. This would be likely to provide conclusive data on the value of using PEG-coated, collagen-based patches to manage anastomotic leakage.
